# Increased Plasticity in Invasive Populations of a Globally Invasive Cactus

**DOI:** 10.3390/plants12183287

**Published:** 2023-09-17

**Authors:** Yohannes B. Tesfay, Annika Blaschke, Nathan Ashley, Liberato Portillo, Alessio Scalisi, Benziane Adli, Juergen Kreyling

**Affiliations:** 1 Institute of Botany and Landscape Ecology, University of Greifswald, 17489 Greifswald, Germany; 2 Odum School of Ecology, University of Georgia, Athens, GA 30602, USA; 3 Department of Botany and Zoology, University of Guadalajara, Guadalajara 44100, Mexico; 4 Department of Energy, Environment and Climate Action, Agriculture, Agriculture Victoria Research, Tatura, VIC 3616, Australia; 5 Department of Biology, Faculty of Nature and Life Sciences, University of Djelfa, Djelfa 17000, Algeria

**Keywords:** phenotypic plasticity, biological invasions, *Opuntia ficus-indica*, non-native populations, greenhouse experiment

## Abstract

Biological invasions pose global threats to biodiversity and ecosystem functions. Invasive species often display a high degree of phenotypic plasticity, enabling them to adapt to new environments. This study examines plasticity to water stress in native and invasive *Opuntia ficus-indica* populations, a prevalent invader in arid and semi-arid ecosystems. Through controlled greenhouse experiments, we evaluated three native and nine invasive populations. While all plants survived the dry treatment, natives exhibited lower plasticity to high water availability with only a 36% aboveground biomass increase compared to the invasives with a greater increase of 94%. In terms of belowground biomass, there was no significant response to increased water availability for native populations, but plants from the invasive populations showed a 75% increase from the dry to the wet treatment. Enhanced phenotypic plasticity observed in invasive populations of *O. ficus-indica* is likely a significant driver of their success and invasiveness across different regions, particularly with a clear environmental preference towards less arid conditions. Climate change is expected to amplify the invasion success due to the expansion of arid areas and desertification. *Opuntia ficus-indica* adapts to diverse environments, survives dry spells, and grows rapidly in times of high-water supply, making it a candidate for increased invasion potential with climate change.

## 1. Introduction

Invasive species successfully establish and spread outside their native range to the detriment of the environment [1]. These biological invasions pose a significant threat to biodiversity and the functioning of ecosystems globally [2,3]. Non-native species are found in biogeographic regions outside their native range, overcoming unfamiliar environmental conditions and selective forces [4]. The rapid adaptation observed in some non-native species upon establishment in new environments has been suggested to explain their successful proliferation and invasive nature [5,6]. Several factors, such as competition for resources, allelopathy, enemy release, climate change, genetic variation and adaptation, contribute to the success of invasive species [7,8,9]. Moreover, a higher degree of phenotypic plasticity is one of the causes that drives an increase in the frequency and extent of plant invasions [10,11,12].

Phenotypic plasticity refers to an organism’s capacity to modify its phenotype in response to environmental changes, enabling it to adapt its observable traits accordingly [13,14]. This adaptive ability of phenotypic plasticity is particularly significant in the context of plant invasions, as it empowers invasive species to acclimatize to novel environments, enhancing their survival and reproductive success. Invasive plants tend to exhibit high levels of plasticity, allowing them to adjust their morphological, physiological, and reproductive traits in different environments [10,15,16].

In addition to the inherently high levels of plasticity observed at the species level, invasive populations can exhibit variations in plasticity when compared to native populations of the same species. Populations of invasive plants in introduced ranges are reported to show greater plasticity than populations in the native range [15,17]. Richards et al. [15] suggest that it is this greater phenotypic plasticity, among other causes, that contributes to the success of invasion by introduced plant species. Accordingly, Kaufman and Smouse [18] report that invasive populations in the introduced ranges of *Melaleuca quinquenervia*, a wetland tree species indigenous to eastern Australia, exhibit higher levels of plasticity in response to different water levels. Similarly, Sexton et al. [17] found significant genetic variation among different ecotypes of *Tamarix ramosissima* populations across its introduced ranges in North America. On the other hand, a common garden drought experiment between the plant populations of *Tanacetum vulgare* from its native range in temperate Europe and the invasive range in North America showed no significant interaction between range and treatment and as a result did not support the evolution of increased phenotypic plasticity in invasive populations [19]. Some invasive species have also been found to create increased intrapopulation genetic diversity once they have established outside of their native ranges [20], thus increasing their survival and adaptability.

Climate change will lead to warmer and drier conditions for large parts of the globe. Consequently, semi-arid and arid tropical regions might expand considerably [21], thereby increasing water stress for the native vegetation. As a cactus, *Opuntia ficus-indica* has the innate ability to survive in dry environments [22,23] and thrive in large areas that become warmer and drier [24]. *Opuntia ficus-indica*, native to Mexico, is a very drought-resistant cactus that is already invasive in arid and semi-arid environments in many parts of the world [25,26,27,28,29]. Recent genetic studies in Mexico have found a very high genetic diversity in this species and highlight the poor understanding of its population structure [30]. High levels of genetic diversity are also described for its invasive populations in Algeria [31], showing that some morphological characteristics were influenced by environmental factors. These morphological responses could potentially become important for understanding the underlying gene-by-environment relationships, referring to the interaction between an individual’s genetic makeup and its environment, which can influence the expression of phenotypes [32]. *O. ficus-indica* is further described as an aggressive invader due to high drought and light tolerance [33]. Likewise, a drought experiment in Italy demonstrated high stress tolerance of locally naturalized *O. ficus-indica* populations, concluding that it will become an important threat to biodiversity conservation in the Mediterranean Basin in the near future [34]. Knowledge of the factors contributing to the invasiveness of *O. ficus-indica* will therefore be important in controlling its spread as the global climate shifts in its favor.

Here, we quantified phenotypic plasticity to water stress in populations from the native (Mexico) and invasive (Africa, Europe) ranges of *O. ficus-indica* in a greenhouse experiment. We hypothesized that phenotypic plasticity in response to water availability is beneficial for invasion success in (semi-)arid environments. Therefore, we anticipated that populations from the invasive ranges would exhibit greater plasticity, displaying improved survival under drought conditions and a more significant increase in biomass production when exposed to higher water availability compared to populations from the native range.

## 2. Results

**Figure 1:**
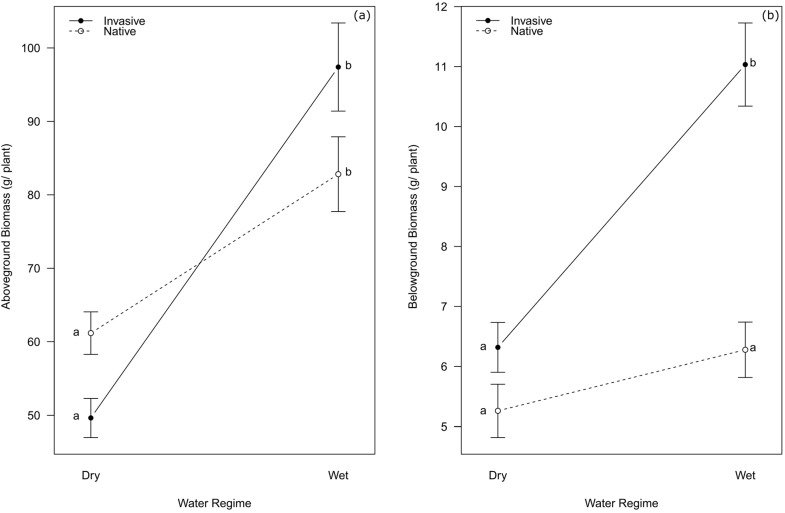
Aboveground (**a**) and belowground (**b**) biomass production (mean ± SE over all populations) of *Opuntia ficus-indica* from the native range (dashed line) and invasive range (solid line) growing under wet and dry regimes. Lowercase letters adjacent to the error bars indicate homogeneous subgroups according to Tukey’s post hoc test. See Appendix A (Figure A1) for the response of individual populations. As a measure of plasticity, ANPP increased 1.4-fold and 2-fold from dry to wet for the native and the invasive populations, respectively; BNPP increased 1.2-fold and 1.8-fold.

*Opuntia ficus-indica* plants from the invasive range showed more plasticity to water availability than plants from the native range in aboveground net primary production (hereafter ANPP) (ANOVA interaction range x water treatment: F = 8.73, *p* = 0.004; Figure 1a). Quantitatively, plants from the native range increased their ANPP from the dry to the wet treatment by only 36%, while the plants from the invasive range increased ANPP by 96%.

The belowground net primary production (hereafter BNPP) of *O. ficus-indica* plants from the native range showed no significant increase between the dry and wet treatments (*p* = 0.501). In contrast, the BNPP of plants from the invasive range exhibited a strong increase of 75% (*p* < 0.001; ANOVA interaction range x water regime: F = 10.32, *p* = 0.002). Moreover, no significant difference was observed between the native and invasive ranges in terms of belowground biomass in the dry treatment (*p* = 0.783), but a significant difference (*p* = 0.034) was observed in the wet treatment, with the invasive range displaying a 76% higher belowground biomass production than the native range under wet conditions (Figure 1b).

**Figure 2:**
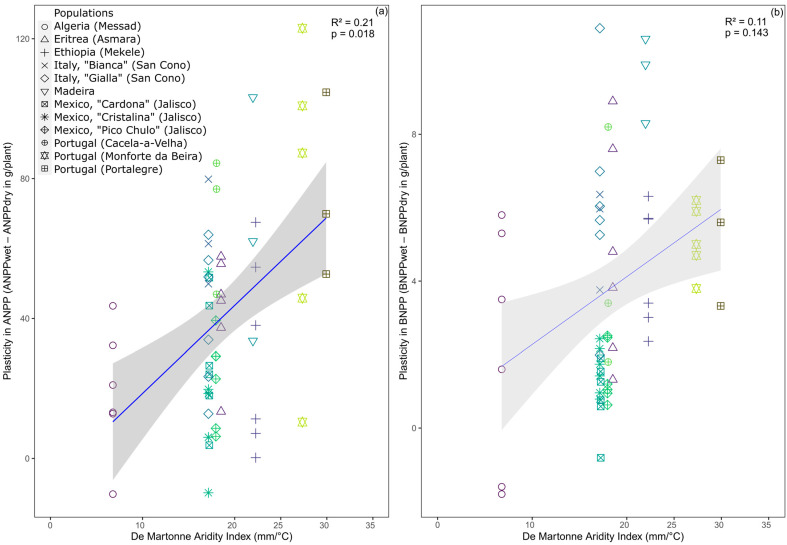
Opportunism to water availability increases with mean annual water availability at the origin of the populations of *Opuntia ficus-indica*. Depicted is the De Martonne aridity index at the populations’ origins (values from 0 to 10 indicate arid climates, 10–20 semi-arid, 20–24 Mediterranean, 24–28 semi-humid, 28–35 humid) and the opportunism as the difference between the aboveground (**a**) or belowground (**b**) net biomass production in the wet versus the dry treatment, i.e., the degree of plasticity in biomass production. The plasticity in net biomass production was calculated by randomly assigning pairs of wet and dry treatments for each population. Symbols show original data points. Linear mixed-effects models with population as a random effect and their 95% CIs are displayed.

All individuals survived the dry treatment. Populations from more humid origins, however, showed stronger responses to additional water than populations from more arid origins for the aboveground biomass (*p* = 0.018; Figure 2a), but there were no significant differences observed in the belowground biomass production (*p* = 0.143; Figure 2b).

## 3. Discussion

The results of our experiment provide evidence for higher levels of plasticity in the response of biomass production to water availability in invasive populations of *Opuntia ficus-indica* compared to native populations. Our findings support previous reports of higher levels of plasticity in plants from invasive populations compared to their native counterparts [10,15,16,17]. The success of invasive species can generally be attributed to two factors regarding the response of fitness traits to environmental variation: (1) their ability to maintain fitness across diverse environments; and (2) their capacity to enhance fitness in favorable environments [35,36,37]. According to our results, *O. ficus-indica* fulfills the second factor more strongly in its invasive than in its native populations. An important point to emphasize is that in addition to the invasive populations of *O. ficus-indica* exhibiting improved water utilization in the wet treatment compared to native populations, all individuals survived the dry treatment. While taking advantage of favorable conditions is beneficial, the capacity to withstand unfavorable conditions, the second factor, is equally important for long-term survival and success. The ability of an organism to increase its own fitness in a changing environment is a competitive advantage over species that lack this ability [38,39]. Plants in general can and have adapted in instances of rapid climate change to maximize their fitness in such variable environments [40]. Invasive plants have proven their capacity to adapt to new environmental conditions and can therefore be expected to also adapt better to changing environmental conditions caused by climate change than presumably less plastic, native species, thus enhancing their advantage [41].

The invasive populations in our study exhibited a greater increase in aboveground biomass under favorable conditions than the native populations. This, coupled with the positive correlation between the environmental conditions at the origin of the populations and the observed increased plasticity (Figure 1a), strongly suggests that plasticity itself is an adaptive trait in *O. ficus-indica*. These results further imply that the species has undergone a rapid adaptive response in plasticity to higher water availability, as the species reached some of its invasive ranges only a few generations ago [28]. The phenotypic changes observed in *O. ficus-indica* within its introduced ranges are indicative of its adaptivity in response to novel environmental conditions [42], and this adaptive potential can be associated with the successful tolerance and invasion of broad geographic areas. While plasticity is certainly not always adaptive, it sometimes is and appears to help *O. ficus-indica* cope with diverse and fluctuating environmental conditions [17,43]. The potential to increase belowground biomass production in response to high water availability appears to be an important and adaptive difference between the invasive and native populations of *O. ficus-indica* (Figure 1b). Cacti are generally recognized for their ability to rapidly develop new roots, enabling efficient water uptake as soon as soil water becomes available [44,45,46]. These rapidly growing roots then disappear again when the soil dries up [47]. We speculate that, in addition to being drought-tolerant [48,49], this ability to dynamically adjust the rooting system has fostered the adaptation of invasive populations to Mediterranean and even sub-humid conditions.

Increased plasticity of *Opuntia ficus-indica* in its invasive populations makes its invasion success even more threatening, as it does not appear to lose its ability to withstand dry periods while making better use of wet conditions. This is supported by the fact that the biomass production of the invasive populations was not significantly lower than that of the native populations in the dry treatment (Figure 1). The ability of *O. ficus-indica* to endure dry periods and exhibit rapid and vigorous growth in response to increasing water availability [33] will significantly contribute to its success in invading new habitats in warming, drying and more fluctuating climate conditions of the future [21]. Here, we have no comparison to growth rates of native species under the same conditions, but we hypothesize that *O. ficus-indica* can tolerate drought better than almost any other species [27,50,51]. Additionally, considering the strong growth observed over the 26-month duration of this experiment, it is likely to effectively compete with native species during wet periods, making it an almost perfect invader in environments characterized by highly fluctuating water availabilities including long and extreme dry periods.

In conclusion, the results of this study demonstrate that *O. ficus-indica* exhibits higher levels of phenotypic plasticity in response to water availability in invasive populations than in native ones. Populations showing a positive relationship between plasticity and water availability at their origin further indicate a quick and beneficial adaptation of plasticity. Conserved water stress tolerance coupled with increased growth under wet conditions in invasive compared to native populations suggest that *O. ficus-indica* will further benefit from climate change, particularly from increased fluctuations in water availability, and continue or even accelerate its spread into native plant communities throughout arid, semi-arid, Mediterranean, and even subhumid ecosystems.

## 4. Materials and Methods

### 4.1. Study Species

*Opuntia ficus-indica* (L.) Mill. is an evergreen perennial cactus that can grow up to 5 m in height. The species has succulent stems that are formed as a sequence of flattened segments called cladodes, which generally have an elliptical base that supports the greatly enlarged and flattened upper portion. *Opuntia ficus-indica* has spines, morphologically corresponding to leaves. Its flowers (5–10 cm in diameter) are sessile and solitary, and the fruits are berries that are 4–8 cm in diameter [52] with an average of 273 seeds per fruit [53]. Nieddu and Chessa [54] found the germination of the *O. ficus-indica* seeds reaching up to 90% in growth chambers with a day/night temperature of 30/20 °C, but only reaching 55% when seeds were kept at room temperature and 43% when seeds were placed outdoors. The seeds are usually dispersed after consumption by humans, birds, and other animals (endozoochoric). Furthermore, vegetative propagation occurs through cladodes readily taking root upon falling to the ground and conspicuous patch formation is an important factor in the persistence of local populations of the plant, although seedling recruitment is essential for expanding the geographic range and establishment in new areas [52].

### 4.2. Experimental Design

**Figure 3:**
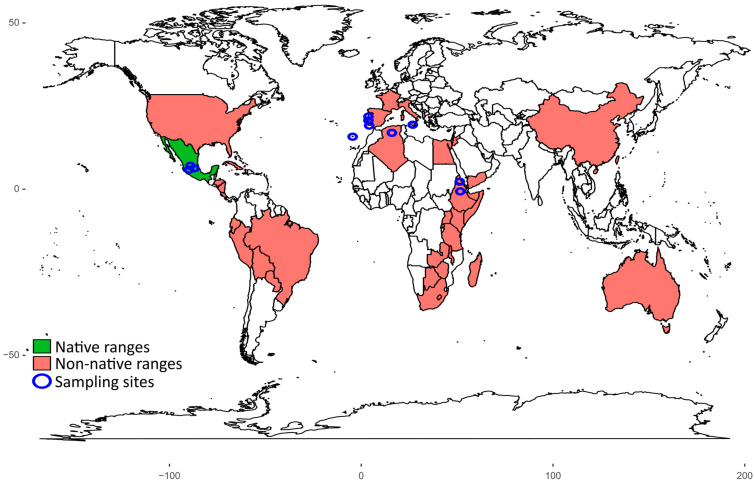
The distribution of *Opuntia ficus-indica*, based upon presence in countries with native range in green (Mexico) and non-native range in light red as reported by Bakewell-Stone [28]. The blue circles indicate the locations where the populations used in this study were collected.

The greenhouse drought experiment was carried out from September 2020 to November 2022 at Greifswald, Germany. We planted the seeds of *O. ficus-indica* from a total of 12 populations (Table 1): three from its native range in Mexico (Jalisco region), three from Africa (Algeria, Eritrea, Ethiopia), one from the island of Madeira off the coast of Africa, and five from Europe (Italy and Portugal), as shown in Figure 3. In Mexico and Italy, different cultivars were collected in order to capture a broad spectrum of genotypes. Taxonomically, the status of the Mexican genotypes “Pico chulo,” “Cristalina,” and “Cardona” is still debated, and some authors see our third genotype as a different species [55,56]. Here, we treat all of them as populations of *O. ficus-indica*, thereby potentially erring towards higher genetic diversity and presumably also phenotypic plasticity in the native range. Consequently, any evidence supporting our hypothesis of higher plasticity in the invasive populations is conclusive, while no difference between the native and non-native populations would be inconclusive.

The cacti were grown in a climate-controlled greenhouse at an average 40% humidity and with a temperature range of 12 °C (for 12 h at night) and 20 °C (for 12 h a day), and windows were set to allow air flow if temperature reached 16 °C (night) or 22 °C (day) for natural cooling. Supplemental lighting, allowing for an additional 115 μmol/m^2^s of photosynthetically active radiation (PAR) to the ambient conditions at pot level, was also provided for 12 h per day from 6:00 to 18:00 using SON-KE 400 high-pressure discharge lamps (DH Licht GmbH, Wülfrath, Germany). The positions of the pots within the greenhouse were frequently interchanged to ensure similar environmental conditions and reduce edge effects or the like.

Plasticity was quantified based on two levels of water availability, depicting dry and wet environments. We measured plasticity by examining how the plant’s phenotype, i.e., above- and belowground biomass, responded to these two environments, comparing the differences in *O. ficus-indica*’s traits between the dry and wet conditions.

**Figure A2:**
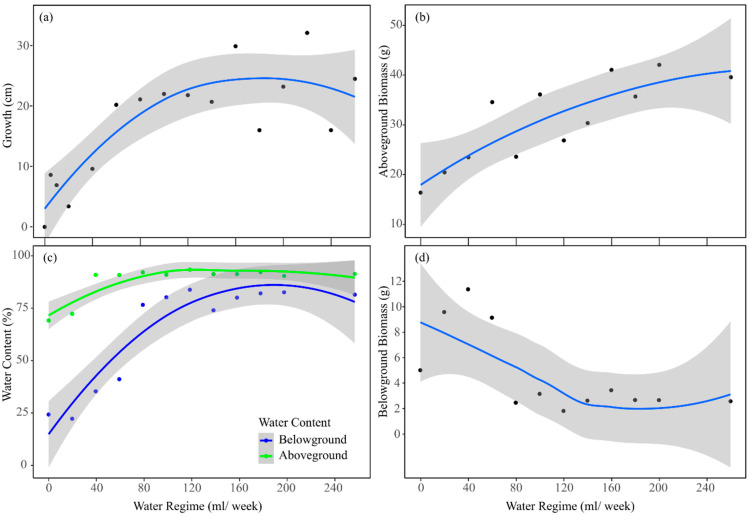
Growth (**a**), aboveground biomass (**b**), water content (**c**), and belowground biomass (**d**) at the end of the drought experiment. Dots are original data points, and lines show the smoothed local polynomial regression fitting (loess). 95% confidence intervals are displayed in grey. This pretrial aimed to observe potentially nonlinear patterns of response variables along the water availability gradient. A powerful tool to answer this kind of question is gradient experiments that maximize treatment levels by minimizing replication [65]. To unravel these response patterns, a graphical analysis was performed. Each response parameter was plotted over the water gradient and smoothed conditional means were calculated by local polynomial regression fitting using the “loess” function, implemented in R [64]. Span was adjusted to produce smooth curves without multiple local extrema based on the assumption that multiple maxima are unlikely in an autecological setting without competition. Confidence intervals (CIs) displayed around the conditional means were used to assess the significance of effects at a level of α = 0.05. The environmental gradient was considered to have a significant effect if a straight horizontal line could not be fitted inside the 95% CI [66].

**Figure A3:**
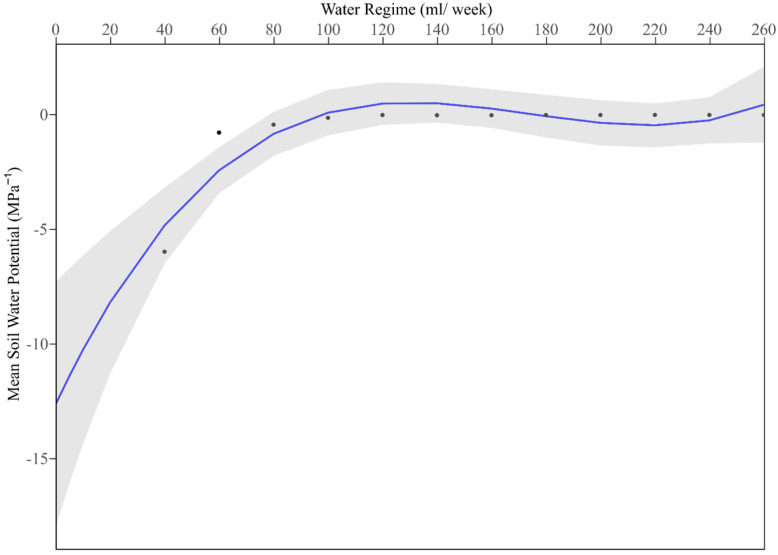
Mean water potential calculated from the entire period of the drought experiment. Dots are the mean data points, and lines show the smoothed local polynomial regression fitting (loess). 95% confidence intervals are displayed in gray. The sensors used to measure the soil water potential (SWP) had different minima and maxima. We were not aware of the limitations when the sensors were placed in the pots for the experiment. The values of the plants from both the extreme sides of the gradient were influenced by the sensors’ lower limit or higher limit, which led to missing data for the driest treatments p0 to p20, where the SWP dropped below the lower limit of the sensors (Teros 21 generation 1:2 MPa^−1^). As a result, these plants’ SWPs are not included in this figure’s SWP parameter, but we extrapolated the spline so that it reaches 0.

The substrate was a mixture of 75% forest soil (loamy sand) and 25% quartz sand. All plants were raised from seeds that were sown in January 2019. After being transplanted into their respective two-liter target pots on 18 September 2020, all plants exhibited similar heights of approximately 40 cm. From a total of 120 pots, the plants from each population (n = 10) were randomly assigned to the two water availability treatments (n = 5), namely, a “wet” treatment that received 160 mL of water twice per week, and a “dry” treatment that received 40 mL of water twice per week. The watering regimes were based on a preliminary pot experiment, exposing 16 plants from Eritrea to a gradient of water availability ranging from no water up to 260 mL twice a week over a period of nine months. The experiment was conducted in the same greenhouse and using the same experimental settings, including pot size, substrate, plant material, and environmental conditions, as the main experiment. All plants survived the pretrial, even though the growth of the plants exposed to drought on the lower end of the gradient, mainly at and below 40 mL, was impeded. The plant exposed to 40 mL, our dry treatment here, showed 80% less aboveground biomass than the wet treatment (160 mL). In a subsequent recovery experiment, the plants that were previously subjected to drought showed rapid aboveground biomass recovery within a span of five days. Additionally, the plant that received the highest amount of water was submerged in a bucket of water for three months and displayed no signs of stress. More details of the pretrial experiment can be found in Appendix B, Figure A2 and Figure A3.

### 4.3. Response Parameters

We quantified ANPP and BNPP at the end of the experiment. We carefully removed each plant from its pot and cut all roots from the stems. The belowground biomass was then gently washed free from the substrate by rinsing it into a coarse sieve so that the substrate was washed away and the roots and rootlets remained. Above and below-ground biomass was dried in separate paper bags for five days at 60 °C and 100% ventilation and the dried samples were subsequently weighed to one decimal point of a gram.

### 4.4. Statistical Analyses

The response variables (ANPP and BNPP) were analyzed by fitting a linear mixed-effects models (lmerTest package, version 3.1-0; [58]), with the explanatory factors being water regime (wet/dry) and range of the species (native/invasive), including their interaction and population identity as a random effect. ANOVA was then applied to extract the significance of the explanatory factors of the mixed model. Single models were run for each response parameter (ANPP and BNPP) for a total of two ANOVA analyses. Tukey’s HSD post hoc tests [59] were used to assess the significance of differences in pairwise comparisons for significant interaction terms.

Moreover, we calculated the De Martonne aridity index [57] as an explanatory factor to identify climatic aridity at the origins of all populations based on temperature and precipitation data for the period 1970–2000 [60]. The De Martonne index is expressed as:

$$I_{DM}=\frac{P}{T_{a}+10}$$

where *P* is the annual amount of rainfall (in millimeters) and *T_a_* is the mean annual air temperature (in degrees Celsius). We tested for a potential relationship between aridity at the origin of the populations and plasticity in growth under different water availability by linear mixed-effects model regression between the De Martonne aridity index as an explanatory variable and the difference between growth in the wet and dry treatments as a response variable. Pairs of wet and dry samples were assigned randomly within each population, and population identity was included as a random effect in the model.

Parametric assumptions were checked for all ANOVA and regression models by examining the diagnostic plots (residuals versus fitted plots for homoscedasticity of the residuals and normal qq-plots for normal distribution of residuals) [61]. When necessary, the ANPP and BNPP datasets were log- or square root-transformed to meet the assumptions. The regressions with the aridity index did not require transformations. For graphical visualizations, the function lineplot.CI in the R package sciplot [62] and ggplot2 [63] were used. All statistical analyses were performed in R version 4.2 [64].

## Figures and Tables

**Table 1:** Populations of *Opuntia ficus-indica* used in this study. De Martonne aridity values from 0 to 10 indicate arid, 10–20 semi-arid, 20–24 Mediterranean, 24–28 semi-humid, and 28–35 humid climate conditions [57].

Range	Area of Origin	Approximate Collection Coordinates	De Martonne Aridity Index (mm/°C)
Non-native	Algeria (Messaad)	34.15212° N, 3.516667° E	7
Non-native	Eritrea (Asmara)	15.23135° N, 38.89911° E	19
Non-native	Ethiopia (Mekele)	14.24886° N, 39.43692° E	22
Non-native	Madeira	32.64838° N, 16.96288° W	22
Non-native	Italy, “Gialla” (San Cono)	37.29390° N, 14.37002° E	17
Non-native	Italy, “Bianca” (San Cono)	37.29390° N, 14.37002° E	17
Non-native	Portugal (Portalegre)	39.27290° N, 7.436978° W	30
Non-native	Portugal (Monforte da Beira)	39.75232° N, 7.281897° W	28
Non-native	Portugal (Cacela-a-Velha)	37.15625° N, 7.546661° W	18
Native	Mexico, “Pico Chulo” (Jalisco)	21.68530° N, 101.4950° W	18
Native	Mexico, “Cristalina” (Jalisco)	22.20615° N, 101.5752° W	17
Native	Mexico, “Cardona” (Jalisco)	21.80851° N, 101.3619° W	17

**Figure A1:**
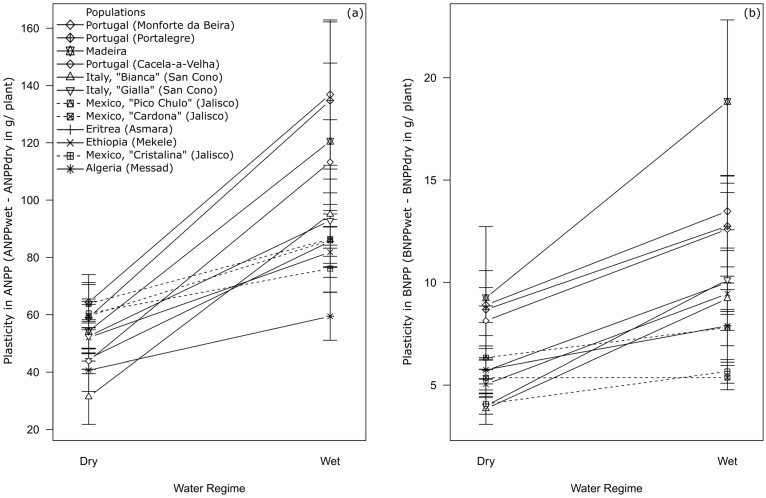
Opportunism to water availability for all populations of *Opuntia ficus-indica* used in the study. Aboveground biomass (**a**) and belowground biomass (**b**) under wet and dry regimes. The solid lines indicate the invasive populations, and the dashed lines indicate the native populations (Mexico) of *O. ficus-indica.* The plasticity in net biomass production was calculated by randomly assigning pairs of wet and dry treatments, respectively, for each population. Linear mixed-effects models with the population as a random effect and their 95% CIs are displayed.

## Data Availability

The datasets generated during and/or analyzed during the current study are available from the corresponding author upon reasonable request.

## Appendix B. Water Tolerance Limits of the Globally Invasive Cactus *Opuntia ficus-indica*

The tolerance limits of *Opuntia ficus-indica* regarding water supply were examined by monitoring growth (Figure A2a), biomass production (Figure A2b,d), water content (Figure A2c) and soil water potential (Figure A3). Sixteen plants from Eritrea were subjected to an irrigation gradient (0 to 260 mL of water per week) for nine months, four were then observed in a recovery experiment for another three months. After the drought and recovery experiment, the plants’ biomass and water content were determined. All plants survived, even though the one subjected to complete drought throughout the trial did not grow. All other plants also showed growth, which was reduced up to a water addition of 60 mL per week and did not change further above that limit. The plants that were previously subjected to drought also showed rapid signs of recovery within five days of the start of the recovery experiment. Concerning drought tolerance, *O. ficus-indica* can survive droughts that last up to nine months and recover quickly. Surprisingly, no sign of reduced performance was found towards the wet end of the gradient. We conclude that *O. ficus-indica* has extremely broad tolerance limits regarding water supply, potentially allowing it to thrive in many regions of the world.
